# A New and Valuable Diagnostic System

**Published:** 1885-12

**Authors:** 


A NEW AND VALUABLE DIAGNOSTIC SYSTEM.—A singular clinical fact in electro-therapeutics is claimed and pointed out by Dr. F. T. Paine, of Comanche, Texas. In his paper, published in this number of the JOURNAL, and to which attention is directed as being of unusual interest and importance, he claims a correspondence, invariably, between functional derangements of the sexual system in male and female, and a loss of sensibility of the mammary regions, and the *ankles; i. e.*, anæsthesia to an electrode of a Farradic battery. If future observation should confirm the correctness of the doctor's statement, and such correspondence should be found, as he says it is an invariable concomitant of either loss of sexual desire, or the reverse—inordinate passion, dyspareunia, etc., and in the male, masturbation or the effects of excessive indulgence, it will constitute a valuable diagnostic sign in those troublesome derangements, and may lead to the solution of a heretofore inexplicable problem, how to cure those obscure and difficult neuroses. The benefits which may follow the discovery of a treatment for dyspareunia or vaginismus, impotence, nymphomania, etc., are incalculable, as the existence of impediments to the marital act has wrecked the happiness of many, and entailed untold misery on scores of men and women.

